# The roles and mechanisms of gut microbiome and metabolome in patients with cerebral infarction

**DOI:** 10.3389/fcimb.2023.1112148

**Published:** 2023-01-25

**Authors:** Wenjun Qian, Miao Wu, Tingting Qian, Chen Xie, Yaxin Gao, Surong Qian

**Affiliations:** Department of Rehabilitation Medicine, The Affiliated Suzhou Hospital of Nanjing Medical University, Suzhou Municipal Hospital, Gusu School, Nanjing Medical University, Suzhou, Jiangsu, China

**Keywords:** cerebral infarction, gut microbiome, metabolome, gut-brain axis, fecal microbiota transplantation (FMT)

## Abstract

As the most common type of stroke, ischemic stroke, also known as cerebral infarction (CI), with its high mortality and disability rate, has placed a huge burden on social economy and public health. Treatment methods for CI mainly include thrombectomy, thrombolysis, drug therapy, and so on. However, these treatments have certain timeliness and different side effects. In recent years, the gut-brain axis has become a hot topic, and its role in nervous system diseases has been confirmed by increasing evidences. The intestinal microbiota, as an important part of the gut-brain axis, has a non-negligible impact on the progression of CI through mechanisms such as inflammatory response and damage-associated molecular patterns, and changes in the composition of intestinal microbiota can also serve as the basis for predicting CI. At the same time, the diagnosis of CI requires more high-throughput techniques, and the analysis method of metabolomics just fits this demand. This paper reviewed the changes of intestinal microbiota in patients within CI and the effects of the intestinal microbiota on the course of CI, and summarized the therapeutic methods of the intervention with the intestinal microbiota. Furthermore, metabolic changes of CI patients were also discussed to reveal the molecular characteristics of CI and to elucidate the potential pathologic pathway of its interference.

## 1 Introduction

Cerebral infarction (CI) is caused by vascular occlusion or arterial stenosis, which is clinically defined as brain tissue injury caused by insufficient blood supply in specific areas, resulting in permanent neuronal damage or even death (Benjamin et al., 2018). CI-induced brain injury is the result of a series of complex neuropathophysiological and neuropathological events, including excitotoxicity, oxidative stress, neuroinflammation, apoptosis, amyloid production, and so on (Kowalski and Mulak, 2019; Pluta et al., 2020; Radenovic et al., 2020). It has been proved that up to 90% of CI cases are related to behavioral factors, including malnutrition, insufficient exercise, smoking and alcohol abuse, and metabolic factors such as diabetes, obesity, hyperlipidemia and hypertension (Li et al., 2019). Identifying potential risk factors and potential pathogenesis that influence the prognosis of CI is of great importance to improve the management and treatment of CI.

The gut-brain axis is a two-way communication system between the brain and the intestine, which regulates intestinal homeostasis and the central nervous system through the neural network, neuroendocrine, immune and inflammatory pathways (Luan et al., 2019; Shaik et al., 2020). Several studies have shown that the gut-brain axis plays an important role in various neurological diseases such as Parkinson’s disease (PD), Alzheimer’s disease (AD), and cerebrovascular diseases (Caputi and Giron, 2018; Megur et al., 2020). Gut microbiota and microbial derived metabolites play a key role in brain function by regulating the gut-brain axis signaling pathway. Recently, it has been shown that intestinal flora imbalance can cause CI and affect the prognosis of CI through inflammatory response and translocation of microorganisms and metabolites caused by intestinal leakage (Chidambaram et al., 2022; Wang et al., 2022a). By exploring the relationship between intestinal microbiota and the pathogenesis and prognosis of CI, feasible strategies will be developed for the prevention and treatment of CI.

The clinical diagnosis of CI is based on neurovascular imaging data or computed tomography, magnetic resonance imaging, ultrasound and angiography (Birenbaum et al., 2011). Unfortunately, incorrect imaging can lead to errors or missed diagnosis, causing delays in receiving adequate treatment and an increased risk of recurrence of CI (Mendelson and Prabhakaran, 2021). Therefore, new biomarker-based tests are still needed to rapidly and accurately diagnose and differentiate CI. Metabolite disturbance is considered to be a key event leading to CI (Floegel et al., 2018). As one of the important components of system biology, metabolomics, together with genomics, transcriptomics, and proteomics, will explain the whole process of life from microscopic DNA molecules to the secretion of small molecule metabolites (Wishart, 2019). Metabolomics can detect the changes of small molecule metabolites after organisms are disturbed, and find their types, quantities and their changing rules (Newgard, 2017). The emergence of this new analytical technique will provide a way to identify key metabolic biomarkers with potential diagnostic and prognostic value in CI.

In this review, the changes of gut microbiota in CI and the mechanism of how they influence the occurrence and development of CI were reviewed, and the literature on human metabolomics in CI, especially amino acid and lipid, was also summarized, aiming to provide new ideas for the potential mechanism and treatment of CI.

## 2 Cerebral infarction

As the second leading cause of death and a major cause of disability worldwide, stroke poses a significant threat to public health (Feigin et al., 2014; Feigin et al., 2017). CI, also known as ischemic stroke (IS), refers to the ischemic necrosis or softening of localized brain tissue caused by a blockage of the blood supply to the brain. CI is the most common clinical type of stroke, accounting for approximately 70%~80%. The mortality rate of CI is about 10%~15%, and the disability rate is high and can be recurrent (Katan and Luft, 2018). According to a 2019 study, there were 13.7 million new CI cases worldwide in 2016, 40% of which were in China. China accounts for 30% of the 5.5 million deaths from CI (Wu et al., 2019).

CI can cause different degrees and types of brain damage, including brain tissue lesions and structural damage, as well as neuronal death and defects (DeLong et al., 2022; Walter, 2022). Patients with disorders including AD, motor abnormalities, low intelligence quotient scores, and numerous cognitive deficiencies have been reported to have clinical symptoms of these sorts of impairments (de Montferrand et al., 2019; Waziry et al., 2020). The recanalization of obstructed arteries and restoration of cerebral blood flow are the ultimate therapeutic goals in the treatment of CI in effort to minimize neuronal damage (Prabhakaran et al., 2015). Nowadays, the most effective treatment for acute CI (ACI) is thrombectomy and thrombolysis (Jolugbo and Ariëns, 2021; DeLong et al., 2022). Although thrombectomy and thrombolysis are effective, not all patients benefit from them without significant side effects and they have high time requirements to take effect (Varona, 2010). Therefore, the diagnosis and treatment of CI require more effective schemes, and more evidence has shown that the role of the gut-brain axis in the diagnosis and treatment of CI needs further exploration.

## 3 Gut microbiome and cerebral infarction

As one of the largest interfaces between the host, the environment, and human antigens, the human gastrointestinal tract contains three types of microorganisms: bacteria, archaea, and eukaryotes. The gut microbiota participates in the operation of the human digestive system, endocrine system, circulatory system, neuroimmune system, and other processes, and plays a vital role in human health and disease processes (Fung et al., 2017; Scheithauer et al., 2020; Wu et al., 2020; Qi et al., 2021; Wu et al., 2022a). Under normal physiological conditions, the gut microbiota maintains a relatively stable state to ensure the health of the body (Wang et al., 2022b). However, changes in the internal and external environment of the body will trigger the imbalance of intestinal microbial flora, which may affect the metabolic and immune response of the host, which can further lead to gastrointestinal dysfunction and various extra-gastrointestinal diseases (de Vos et al., 2022).

In recent years, the concept of brain-gut axis has been proposed, pointing out that the brain-gut axis is a two-way neural pathway connecting the brain, central nervous system (CNS), enteric nervous system, and autonomic nervous system (Luan et al., 2019). After the brain integrates the relevant external information and the body, it can act directly on the smooth muscle cells of the gastrointestinal tract, or transmit regulatory information to the neural plexus of the gastrointestinal tract along the autonomic nerve and neuroendocrine system, so as to perform the operation of the gut-brain axis (Agirman et al., 2021; Mayer et al., 2022). Intestinal microbial flora imbalance has been implicated to the onset and progression of several neurological diseases, including PD, AD, multiple sclerosis, depression, and so on, according to an increasing number of studies (Sun and Shen, 2018; Simpson et al., 2021; Chen et al., 2022; Xie et al., 2023). More research is also pointing to the pivotal function gut microbiota plays in CI.

### 3.1 Changes of gut microbiota in cerebral infarction

CI is closely related to changes in the composition of the intestinal microbiota (**Table 1**). Analysis of the proportion of various components of the intestinal microbiota in patients is helpful in identifying possible symptoms of CI as early as possible. Yamashiro et al. analyzed the fecal gut microbiota composition of CI patients and control subjects. The results showed that CI was closely associated with an increase in *Atopobium* cluster and *Lactobacillus ruminis*, and a decrease in the *Lactobacillus sakei* subgroup (Yamashiro et al., 2017). A prospective observational study of short-chain fatty acid (SCFA) profiles in patients with ACI in China showed that SCFAs-producing bacteria (*Roseburia*, *Bacteroides*, *Lachnospiraceae*, *Faecalibacterium*, *Blautia*, and *Anaerostipes*) were less abundant in ACI patients while *Lactobacillaceae*, *Akkermansia*, *Enterobacteriaceae*, and *Porphyromonadaceae* were overgrown, which reflected the dysregulation of the intestinal microbiota in ACI patients (Tan et al., 2021). However, another study showed the opposite. The analysis of the gut microbiota of ACI patients and healthy controls by Li et al. showed an increase in SCFAs producing bacteria in patients, including *Odoribacter*, *Akkermansia*, *Ruminococcaceae_UCG_005*, and *Victivallis* (Li et al., 2019).

**Table 1:** Changes of gut microbiota in cerebral infarction.

Authors	Gut microbiota	Patients	Outcomes	References
Li et al.	*Odoribacter, Akkermansia, Ruminococcaceae_UCG_005*, and *Victivallis*	30 CI patients	Similar microbial α-diversity between CI patients and HCs. More SCFAs producer including *Odoribacter, Akkermansia, Ruminococcaceae_UCG_005* and *Victivallis* in CI patients.	(Li et al., 2019)
Yamashiro et al.	*Atopobium* cluster and *Lactobacillus* ruminis	40 CI patients	Increased numbers of the *L. ruminis* subgroup Decreased counts of the *L. sakei* subgroup	(Yamashiro et al., 2017)
Tan et al.	Lactobacillaceae, Enterobacteriaceae and Porphyromonadaceae	140 ACI patients	Decreased counts of SCFA‐producing bacteria (*Roseburia*, *Bacteroides*, *Lachnospiraceae*, *Faecalibacterium*, and so on). Increased numbers of Lactobacillaceae, *Akkermansia*, Enterobacteriaceae and Porphyromonadaceae in ACI patients.	(Tan et al., 2021)
Karlsson et al.	*Collinsella, Roseburia* and *Eubacterium*	12 patients with symptomatic atherosclerotic plaques	Enriched *Collinsella* and decreased *Eubacterium* and *Roseburia* in symptomatic atherosclerotic plaque.	(Karlsson et al., 2012)

CI, cerebral infarction; ACI, acute cerebral infarction; HCs, health controls; symptomatic atherosclerotic plaques, plaques from patients who had undergone carotid endarterectomy for minor ischemic stroke, transient ischemic attack or amaurosis fugax.

Atherosclerosis is a possible cause of CI. The gut microbiota analysis of patients with atherosclerotic plaque who had undergone carotid endovascular resection for mild CI, transient ischemic attack, or transient amaurosis leaurosis showed that *Collinsella* enriched in symptomatic atherosclerotic plaque, while *Eubacterium* and *Roseburia* decreased. Patients’ metagenome is rich in genes associated with peptidoglycan biosynthesis, suggesting that intestinal metagenomic increased peptidoglycan production may promote symptomatic atherosclerosis by primes the innate immune system and enhances neutrophilic function (Karlsson et al., 2012). Although studies have demonstrated a relationship between clot histology and CI, few cases involving *Candida* have been reported. Clot analysis of a patient with ACI showed a notable presence of *Candida albicans* (Walker et al., 2019).

### 3.2 Possible mechanisms by which gut microbiota alters cerebral infarction

The inflammatory response to sterile tissue injury is a key pathophysiology of organ-specific injury, including CI (Chamorro et al., 2012). Previous studies have shown that pro-inflammatory Th1, Th17, and γδ T cells are associated with increased inflammatory damage and poor prognosis, while Treg cells suppress neuroinflammatory responses to brain injury (Gelderblom et al., 2012; Liesz et al., 2013). The evidence suggested that the gut microbiota is a key regulator of T cell homeostasis and is closely related to the maturation of the immune system and the coexistence of the maintenance host and microbes. Benakis et al. showed that intestinal dysbiosis affects Treg and IL-17 γδT cells and found that intestinal lymphocyte migration to the ischemic brain may be associated with increased infarct volume, while antibiotic-induced changes in the intestinal microbiota significantly reduced ischemic brain injury in mice (Benakis et al., 2016). Singh et al. observed dysbiosis of the gut microbiota by pressure-mediated intestinal palsy after CI, which in turn was causally associated with changes in T cell homeostasis, induction of the pro-inflammatory response, and worsening of stroke outcomes. Fecal microbiota transplantation (FMT) will contribute to improved stroke outcomes (Singh et al., 2016).

In addition, gut-derived damage-associated molecular patterns (DAMPs) and cytokine storms may affect the outcome of CI by regulating CNS antigen specific immune response. Tascilar et al. found that some intestinal bacteria in a mouse model of occlusion of the middle cerebral artery were translocated from the gut to the blood prior to the onset of the symptoms of CI. In addition, intestinal bacteria were transferred from the gut to the testes and other organs after CI in mice, and systemic inflammatory reactions occurred in some models later, causing post-stroke infection. However, the exact mechanism involved in this process remains unclear and needs further study (Tascilar et al., 2010). The gut microbiota of young mice with CI was altered, and in aged mice, the ratio of the two main bacterial phylums *Firmicutes* to *Bacteroides* phylum (F:B) increased approximately 9-fold compared to young mice, indicating dysbiosis. After altering the microbiota of young mice by fecal transplant gavage to increase their F:B ratio by about 6 times, cytokine levels were found to be significantly increased (Spychala et al., 2018). As a result, intestinal dysbiosis can create a vicious pro-inflammatory cycle that reduces the prognosis after stroke.

### 3.3 Therapeutic potentials of gut microbiota in cerebral infarction

Regulation of the gut microbiota by antibiotics is a proven strategy, which can remove or prevent bacterial colonization in the human body, greatly affect the composition of the gut microbiota and reduce its biodiversity (Angelucci et al., 2019). The feasibility of alleviating CI by regulating intestinal microbial imbalance through antibiotics is being verified by various studies. Benakis et al. treated male mice with either a combination of antibiotics or a single antibiotic, respectively, and found that mice treated with a combination of antibiotics showed a significant reduction in infarct volume during the acute phase of stroke, while a single antibiotic treatment with ampicillin or vancomycin also reduced infarct volume and improved motor sensory function within 3 days after stroke (Benakis et al., 2020). Chen et al. applied antibiotics to CI rats, which decreased the α diversity of the intestinal microbiome, infarcted volume, and significantly increased acetic acid and valeric acid levels in ischemic rats (Chen et al., 2019). However, to date, there is no clear evidence to support prophylactic antibiotic therapy in the first hours after ACI to control ecological dissonance (Ulm et al., 2017). Two large randomized controlled phase III clinical trials did not show an improvement in results after ACI with prophylactic administration, and according to the results, antibiotics are not recommended for the prevention of CI (Kalra et al., 2015; Westendorp et al., 2015).

FMT is defined as the transfer of healthy gut bacteria *via* donor stool to a patient, aiming to obtain therapeutic benefits by directly altering or normalizing the gut microbiota of recipients (Khoruts and Sadowsky, 2016). Recent studies have confirmed FMT as a possible strategy to regulated intestinal ecological disorders in patients with neuropsychiatric disorders and patients affected by CI (Evrensel and Ceylan, 2016). Wang et al. performed FMT in a CI mouse model to verify the influence of different sexes microbiomes on the prognosis of CI. The results showed increased survival, reduced infarct size, improved performance in behavioral tests, increased release of beneficial metabolites, and reduced levels of inflammation in mice receiving the female gut microbiome. In contrast, mice that received the male microbiome were less effective in preventing brain damage and restoring neural function (Wang et al., 2022c). Chen et al. showed that FMT intervention significantly changed the intestinal microbiome composition of CI, reduced pathogenic bacteria and increased beneficial bacteria, and thus reduced neurological function damage, eliminated cerebral edema and reduced infarct volume. Further studies have shown that regulating SCFA levels such as isobutyric acid, butyric acid, and isovalerate may be the mechanism by which FMT alleviates CI (Chen et al., 2019).

The benefits of probiotics on host health have been extensively studied and clearly defined (Hill et al., 2014). Several studies have confirmed the efficacy and mechanism of probiotics in preventing CI by regulating the composition of the gut microbiota, improving intestinal barrier function, and regulating local and systemic immunity. Rodent models have verified the roles of *Lactobacillus*, *Clostridium butyricum*, and *Bacillus licheniformis* in CI (Fang et al., 2022). Through the regulation of TLR-4/NF-kappa B signaling, *Lactobacillus* reduced the extent of cerebral infarction, lowered oxidative stress, and blocked the death of brain cells, which improved neurobehavioral scores (Wanchao et al., 2018). After pretreatment with *C. butyricum*, butyrate content in the brain increases significantly, regulating CNS function and alleviating CI (Sun et al., 2016). “Synbiotics” is a combination of probiotics and matrices (Swanson et al., 2020). As opposed to inulin alone or SCFA-producing bacteria alone, Lee et al. discovered a synergistic impact of inulin and SCFA-producing bacteria, which improved neurodeficiency scores and behavioral outcomes in mice following stroke (Lee et al., 2020).

## 4 Metabolomics and cerebral infarction

Metabolomics is one of the important components of system biology, which is the science of studying the type, quantity and change law of metabolites after an organism is perturbed (Johnson et al., 2016; Wu et al., 2022b). Metabolomics can reflect a series of biological events that occur in a pathophysiological process by revealing the trajectory of the overall metabolism under the influence of internal and external factors (Tian et al., 2022; Xiang et al., 2022). Studying CI through metabolomics can reveal its molecular signature and elucidate potential pathological pathways in which diseases are disturbed (**Table 2**).

**Table 2:** Metabolic biomarkers in cerebral infarction.

Authors	Metabolic biomarkers	Subjects and specimens	Outcomes	References
Kimberly et al.	Valine, leucine and isoleucine	Plasma from patients with CI	Reduction in the BCAAs (valine, leucine, isoleucine) in human plasma compared to HCs, which correlated with poor neurological outcome.	(Kimberly et al., 2013)
Zheng et al.	Glutamate and glutamine	Plasma from patients with CVDs (including CI)	Increased stroke risk with baseline Glu levels; Decreased stroke risk with baseline levels of Gln and Glu.	(Zheng et al., 2016)
Wang et al.	Oleic acid, linoleic acid, L-glutamine, L-arginine, and L-proline	Serum samples from patients with AIS	Higher levels of oleic acid, linoleic acid, arachidonic acid, and so on; Lower levels of L-glutamine, L-arginine, and L-proline.	(Wang et al., 2020)
Hu et al.	Tyrosine, citrulline, proline, C4/C2 and alanine	Dried blood spot samples from patients with CI	Elevated levels of tyrosine, citrulline and proline, implying a decrease in neuronal autophagy, apoptosis, and platelet dysfunction; Elevated level of C4/C2 and alanine, which is closely related to neuronal autophagy.	(Hu et al., 2016)
Ye et al.	Phospholipids, sphingolipids, and glycerides	Serum from patients with CI	Disturbed PL, SL and glycerides metabolism in the serum of CI patients.	(Ye et al., 2022)
Sheth et al.	Sphingolipids	Plasma from mouse model of ACI	Dramatic increase in SL levels in the stroke compared to sham animals; The majority of the top performing species were SM and Cer.	(Sheth et al., 2015)
Grandizoli et al.	Phosphatidylcholine, phosphoethanolamine and SM	Plasma from patients with CI	Phosphorus-containing compounds can be considered as important biomarkers in the investigation of CI.	(Grandizoli et al., 2014)
Jung et al.	Lactic acid, pyruvate, glycolic acid and formate	Plasma and urine from patients with CI	Increased excretion of lactic acid, pyruvate, glycolate and formate in plasma; Decreased levels of citrate, hippurate and glycine in urine.	(Jung et al., 2011)

CI, cerebral infarction; ACI, acute cerebral infarction; CVDs, cardiovascular diseases; BCAAs, branched chain amino acids; HCs, health controls; PL, phospholipid; SL, sphingolipid; SM, sphingomyelins; Cer, ceramides.

### 4.1 Amino acid metabolism and cerebral infarction

At the cellular level, excitotoxicity is a key cellular mechanism for cerebral ischemic injury (Rothman and Olney, 1986). This is triggered by metabolic homeostasis failure and secreted metabolites including glutamate, glycine, D-serine, and polyamines. Significant glutamate accumulation and down-regulation of glutamine ratios have been shown in ACI mouse models and in patients with cardiovascular diseases, including CI (Kimberly et al., 2013; Zheng et al., 2016).

Branched chain amino acids (BCAAs) are components of the glutamate/glutamine cycle between astrocytes and neurons and are essential for signaling in excitatory neurons (Salcedo et al., 2021). Metabolomic analysis of plasma in patients with mild and moderate ACI has found that reduced concentrations of BCAAs, including leucine, isoleucine, and valine, are associated with stroke severity and a worse prognosis (Kimberly et al., 2013). Wang et al. obtained the serum metabolic profile of stroke patients using the untargeted metabonomic method, and found that there were metabolic disorders in patients with ACI. Compared to HCs, the levels of 4-hydroxyproline, L-glutamine, L-arginine, and L-proline in patients with ACI were lower, revealing an increased risk of cerebrovascular diseases (Wang et al., 2020). Hu et al. directed injection liquid crystal mass spectrometry analysis of dried blood spots in patients with CI showed a significant increase in the ratio of tyrosine, citrulline, and proline, which also implied a decrease in neuronal autophagy, apoptosis, and platelet dysfunction (Hu et al., 2016).

### 4.2 Lipid metabolism and cerebral infarction

As a subset of metabolomics, lipidomics shows functions similar to metabolomics (Lam et al., 2017). In recent years, the analysis of lipids has been greatly improved by matrix-assisted laser mass spectrometry and other techniques (Adibhatla et al., 2006). Several studies have shown that lipids play an important role in the occurrence and development of diseases. Lipids cross the blood-brain barrier more easily than proteins and enter brain cells, and the high abundance of polyunsaturated fatty acids in brain lipids makes the brain more susceptible to oxidative stress than most tissues and organs (Hamilton et al., 2007).

Animal models and human studies related to CI have shown that abnormal lipid metabolism is closely related to the prediction and prognosis of CI, including phospholipids, sphingolipids (SLs), and glycerides (Ye et al., 2022). Sheth et al. measured and evaluated the changes in plasma SL concentration in the mouse model of ACI, and the results showed that SLs were highly enriched in the brain, among which the most obvious changes were ceramide and sphingomyelin (SM), and the concentration of multiple SLs in the brain and plasma differed by more than 1000 times. The feasibility of this targeted lipid analysis was verified in patients with ACI (Sheth et al., 2015). Human serum metabolomics based on nuclear magnetic resonance was performed on blood phosphorus in patients with CI, and it was found that serum phosphatidylcholine, phosphoethanolamine and SM levels were lower in CI patients compared with healthy individuals (Grandizoli et al., 2014). Therefore, phosphorus-containing compounds in human serum can be considered important biomarkers in CI research.

### 4.3 Other metabolites and cerebral infraction

Oxidative stress in cerebral ischemia is caused by the excessive production of oxygen derivatives and metabolic dysfunction. Stroke causes heterogeneous changes in tissue oxygenation and produces lactic acid as the end product, thus making the cytoplasmic environment acidic (Liu et al., 2004). Excess protons convert oxygen into hydrogen peroxide and reactive hydroxyl radicals (Shin et al., 2020). Jung et al. found that the levels of lactic acid, pyruvate, glycolic acid and formate in plasma and urine of patients with cerebral ischemia increased, while the levels of glutamine and methanol decreased, which reflected the oxidative stress state of cerebral ischemia (Jung et al., 2011).

In individuals with cerebral ischemia, oxidative stress and blood-brain barrier damage can also aggravate the inflammatory response (Sidorov et al., 2019). Glial cells, neutrophils, monocytes, and lymphocytes are seen in higher concentrations with the activation of inflammation, along with pro-inflammatory cytokines and metabolites that are connected to inflammation (Shin et al., 2020). The increase of C4/C2 could indicate the deficiency of short-chain acyl-coA dehydrogenase. This is closely related to the inactivation of peroxisome proliferator-activated receptor alpha, which may reflect platelet dysfunction. The increase of alanine level is believed to be related to the increase of carnosin content in blood samples of cerebral ischemia patients, and is closely related to neuronal autophagy (Hu et al., 2016).

## 5 Conclusions

**Figure 1:**
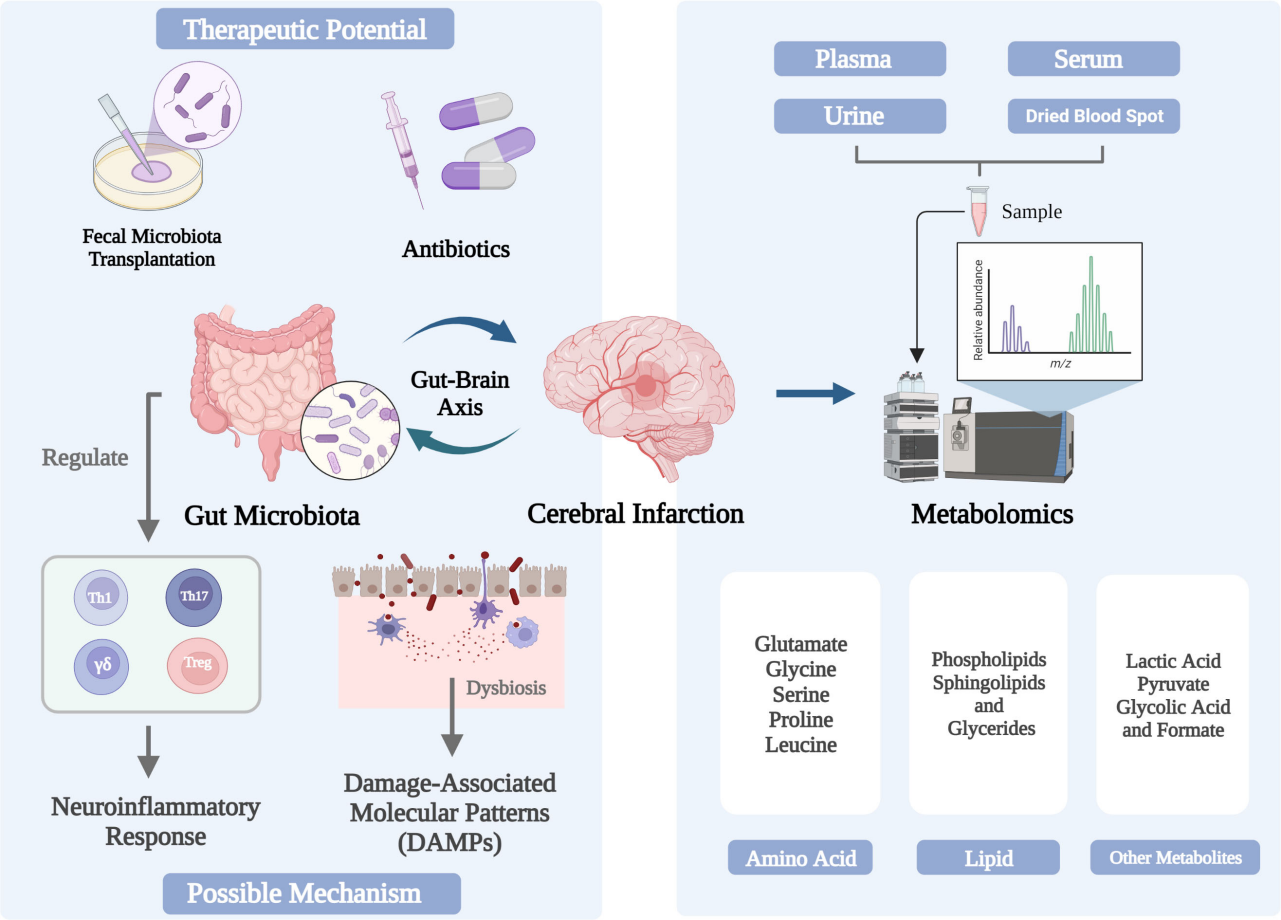
Changes and in the gut microbiome and metabolome associated with cerebral infarction and their triggering mechanisms.

The physiological functions of brain and intestine are closely related. Intestinal flora can interact with the brain through various mechanisms. The imbalance of intestinal flora will promote the occurrence of CI and is closely related to the prognosis, while CI may aggravate the ecological imbalance of intestinal flora. Intestinal microorganisms significantly affect CI through neuroinflammation and other pathways (**Figure 1**). There is evidence that the cohabitation of the maintenance host and microorganisms, as well as the gut microbiota, are directly associated to immune system development and the regulation of T cell homeostasis. Additionally, gut microbiota may influence how CI turns out by gut-derived DAMPs and cytokine storms. Nowadays, CI treatment by altering the intestinal microbiota, including FMT, antibiotics and probiotics, still has some limitations, and more research is needed. Future research may focus on the mechanisms of microbe-host interactions, the use of high-throughput sequencing of gut microbial genomes, and the development of drugs based on these data. Therefore, as the omics closest to the phenotype, metabolomics emerged at the historic moment and has become an important breakthrough in scientific research. Excitotoxicity, which is brought on by the loss of metabolic homeostasis and specific types of amino acids, is a crucial biological mechanism for cerebral ischemia damage at the cellular level. Lipids are able to infiltrate brain cells and bridge the blood-brain barrier more readily than proteins. Additionally, the brain is more vulnerable to oxidative stress than most other tissues and organs due to the high concentration of polyunsaturated fatty acids in brain lipids. The analysis of amino acids, lipid and other metabolites in CI patients is helpful to discover new biomarkers of CI, new pathophysiological mechanisms and innovative therapeutic methods. To translate these studies into clinical applications, complete prospective and longitudinal studies are essential.

## Author contributions

WQ, MW, and SQ had the idea for the article; CX and YG performed the literature search and data analysis; SQ drafted and critically revised the work. All authors contributed to the article and approved the submitted version.
